# Mortality after Inpatient Treatment for Severe Pneumonia in Children: a Cohort Study

**DOI:** 10.1111/ppe.12348

**Published:** 2017-03-20

**Authors:** Moses M. Ngari, Greg Fegan, Martha K. Mwangome, Mwanajuma J. Ngama, Neema Mturi, John Anthony Gerard Scott, Evasius Bauni, David James Nokes, James A. Berkley

**Affiliations:** ^1^ KEMRI/Wellcome Trust Research ProgrammeKilifiKenya; ^2^ The Childhood Acute Illness & Nutrition (CHAIN) NetworkNairobiKenya; ^3^ Swansea Trials UnitSwansea University Medical SchoolSwanseaUK; ^4^ London School of Hygiene & Tropical MedicineLondonUK; ^5^ School of Life SciencesUniversity of WarwickCoventryUK; ^6^ Centre for Tropical Medicine & Global HealthUniversity of OxfordOxfordUK

**Keywords:** children, severe pneumonia, post‐discharge, mortality

## Abstract

**Background:**

Although pneumonia is a leading cause of inpatient mortality, deaths may also occur after discharge from hospital. However, prior studies have been small, in selected groups or did not fully evaluate risk factors, particularly malnutrition and HIV. We determined 1‐year post‐discharge mortality and risk factors among children diagnosed with severe pneumonia.

**Methods:**

A cohort study of children aged 1–59 months admitted to Kilifi County Hospital with severe pneumonia (2007–12). The primary outcome was death <1 year after discharge, determined through Kilifi Health and Demographic Surveillance System (KHDSS) quarterly census rounds.

**Results:**

Of 4184 children (median age 9 months) admitted with severe pneumonia, 1041 (25%) had severe acute malnutrition (SAM), 267 (6.4%) had a positive HIV antibody test, and 364 (8.7%) died in hospital. After discharge, 2279 KHDSS‐resident children were followed up; 70 (3.1%) died during 2163 child‐years: 32 (95% confidence interval (CI) 26, 41) deaths per 1000 child years. Post‐discharge mortality was greater after admission for severe pneumonia than for other diagnoses, hazard ratio 2.5 (95% CI 1.2, 5.3). Malnutrition, HIV status, age and prolonged hospitalisation, but not signs of pneumonia severity, were associated with post‐discharge mortality. Fifty‐two per cent (95% CI 37%, 63%) of post‐discharge deaths were attributable to low mid‐upper arm circumference and 11% (95% CI 3.3%, 18%) to a positive HIV test.

**Conclusions:**

Admission with severe pneumonia is an important marker of vulnerability. Risk stratification and better understanding of the mechanisms underlying post‐discharge mortality, especially for undernourished children, are needed to reduce mortality after treatment for pneumonia.

Worldwide, pneumonia is the leading cause of death for children <5 years and accounted for approximately 1.3 million deaths in 2011.^1^, ^2^ Several studies have examined inpatient treatment failure and death amongst children with severe pneumonia.^3^, ^4^, ^5^, ^6^ However, deaths are known to also occur after discharge from hospital. A systematic review of paediatric post‐discharge mortality in resource‐poor countries, identified 13 studies.^7^ Only three of these studies specifically reported outcomes among children admitted with pneumonia: from The Gambia (*n* = 118), Bangladesh (*n* = 162), and Tanzania (*n* = 666).^8^, ^9^, ^10^ Two of the studies excluded children with severe malnutrition or other co‐morbidities,^9^, ^10^ and one examined the effect of HIV prior to widespread availability of antiretroviral therapy.^10^

Since the systematic review in 2013,^7^ three studies reporting post‐discharge mortality among children admitted with pneumonia have been published.^11^, ^12^, ^13^ Two of these studies included hospitalised children with a diagnosis of pneumonia, but not necessarily severe pneumonia according to World Health Organization (WHO) guidelines, from The Gambia (*n* = 2725)^11^ and Uganda (*n* = 389).^12^ Only the Ugandan study^12^ examined the effect of HIV status. One other study, from Bangladesh (*n* = 369)^13^ used WHO diagnostic criteria for severe pneumonia, but only included HIV‐uninfected children with Severe Acute Malnutrition (SAM). The duration of follow‐up varied between 3 months and more than 12 months, and none adequately reported loss to follow‐up or expressed mortality as an incidence rate. Thus, current knowledge of post‐discharge mortality following treatment from severe pneumonia, and its risk factors, is limited.

We aimed to determine 1‐year post‐discharge mortality rate and its risk factors amongst children consecutively admitted to a rural Kenyan hospital with syndromically diagnosed severe pneumonia.^14^

## Methods

### Study setting

This was an observational cohort study. The study was conducted at Kilifi County Hospital (KCH) in a rural area on the Kenyan coast. *Haemophilus influenzae* type b and pneumococcal conjugate vaccines were introduced in November 2001 and February 2011, respectively.^15^, ^16^ The antenatal HIV prevalence is 4.9%.^17^ KCH provides inpatient and outpatient services for HIV and malnutrition. Exposures were clinical, laboratory, and demographic features at hospitalisation. The primary outcome was death within 1 year after discharge from hospital. The study was approved by the Kenya Medical Research Institute (KEMRI) National Ethical Review Committee (SCC 2778).

### Study population

We analysed systematically collected surveillance data from all children aged 1 to 59 months admitted to KCH between January 2007 and December 2012, and followed up those resident in the Kilifi Health and Demographic Surveillance System (KHDSS) until April 2014. We initially included non‐KHDSS residents in order to better understand the characteristics and generalisability of the children who were followed up in the context of all children served by the hospital. Trained clinicians provided care according to WHO guidelines.^14^

### Clinical definitions and care

Severe pneumonia was defined by WHO (2005)^14^ as cough or difficulty breathing plus either lower chest wall indrawing or inability to breast feed/drink/vomiting everything, impaired consciousness, central cyanosis or peripheral oxygen saturation <90% by pulse oximetry (Nelcor USA).^3^, ^14^ Impaired consciousness was defined as ‘prostration’ (inability to sit unassisted at ≥1 year; inability to drink or breast feed at <1 year) or ‘coma’ (Blantyre coma score ≤2). We classified children as having the syndromes of severe or very severe pneumonia, hereafter called ‘severe pneumonia’ (including children who had an additional diagnosis), or not having severe pneumonia.

As previously described,^18^, ^19^ trained clinical assistants measured Mid‐upper arm circumference (MUAC) with a non‐stretch measuring tape (TALC, St Albans, UK), weight with an electronic scale (Seca, Birmingham, UK) that was checked weekly for consistency, and length using a measuring board of standard United Nation Children's Fund (UNICEF) design (for children younger than 2 years or those who could not stand) or height using a wall‐mounted stadiometer (Seca, Birmingham, UK). Inpatient management of SAM was based on one or more of weight‐for‐length z‐score, MUAC or the presence of kwashiorkor, and followed WHO guidelines.^14^ Children with SAM were discharged to therapeutic and/or supplementary feeding programmes as per national guidelines.

HIV testing using two rapid antibody tests, Determine (Inverness Medical, Florida, USA) and Unigold (Trinity Biotech, Bray, Ireland), was systematically offered to all paediatric admissions according to national guidelines.^20^ Families of patients with a positive test were counselled and referred for comprehensive care. Blood culture was systematically undertaken by methods previously published.^18^ We defined severe anaemia as haemoglobin <5 g/dL. As part of an ongoing study, nasopharyngeal specimens were systematically collected from children with severe pneumonia and tested for respiratory syncytial virus (RSV) using a Direct Immunofluorescent Antibody Test (Chemicon, Temecula, CA).^21^, ^22^ Nasopharyngeal samples were not collected in a high proportion of the most severely ill children (i.e., those in the high dependency ward), as previously reported.^21^ RSV was the only viral pathogen more frequently detected in severe pneumonia compared to controls without pneumonia.^21^

Follow‐up after discharge was done through the KHDSS. Within the KHDSS, the community of approximately 262 000 residents in an area of 891 km^2^ immediately surrounding the hospital has been allocated unique identifiers that are matched when patients are admitted to hospital. Households are enumerated by trained fieldworkers for vital events every 4 months, as described elsewhere.^17^

### Statistical analysis

Participants were stratified either as residents of KDHSS or non‐residents. Outlying anthropometric z‐score values were excluded if their values were ±6 from the median z‐score for each anthropometric parameter. This method differs from data cleaning methods commonly applied to community surveys (usually ±3 z‐score from the observed survey mean)^23^ because very low values were typically confirmed as genuine within this range amongst this population of children who were sick enough to be admitted to hospital.

To determine post‐discharge mortality, we used KDHSS census data from January 2007 to April 2014. The time at risk considered was from discharge to 365 days later, or the date of out‐migration or death. We performed a single discharge analysis, where only the first admission during the study period was considered. Data from children with missing outcomes were excluded. Absent HIV antibody and RSV test data were analysed as separate categories as they were assumed not to have been missed randomly. Post‐discharge mortality was reported as the incidence rate per 1000 child‐years of observation.

Variables were investigated as potential risk factors for post‐discharge mortality based on previous work.^3^, ^24^ A binary variable for previous admission was used in the analysis since only 0.9% (22/2461) of KHDSS‐residents who were discharged alive had >1 prior admission. We plotted Kaplan—Meier curves and used Cox proportional hazards models to test associations with post‐discharge mortality, retaining all variables in the multivariable model. We tested for interactions by comparing models using likelihood‐ratio tests. Survival distributions were compared using the log‐rank test. Goodness of fit was assessed using correlation coefficient, R‐squared and Akaike information criterion.

Because of the anticipated role of undernutrition in driving mortality, that stunting as well as thinness may be associated with increased mortality risk, and debate over which indices are most informative,^25^ we built three multivariable models using different anthropometric parameters: (i) using MUAC, which captures elements of age, is less affected by dehydration than weight‐based indices and appears to be more reliable than calculated length‐based z‐ scores in young children;^25^, ^26^, ^27^ (ii) using both of the traditional indicators of acute and chronic malnutrition, wasting (weight‐for‐height z‐score) and stunting (length or height‐for‐age z‐score); and (iii) using weight‐for‐age z‐score, which captures all factors affecting bodyweight.

Internal validation was done by calculating bootstrapped area under the receiver operating curve (AUROC) and bias‐corrected 95% confidence intervals estimated using a probit model, resampled 200 times (with replacement). Population attributable fractions (PAF) were calculated^28^ and adjusted for age, gender, and HIV status.

## Results

**Figure 1:**
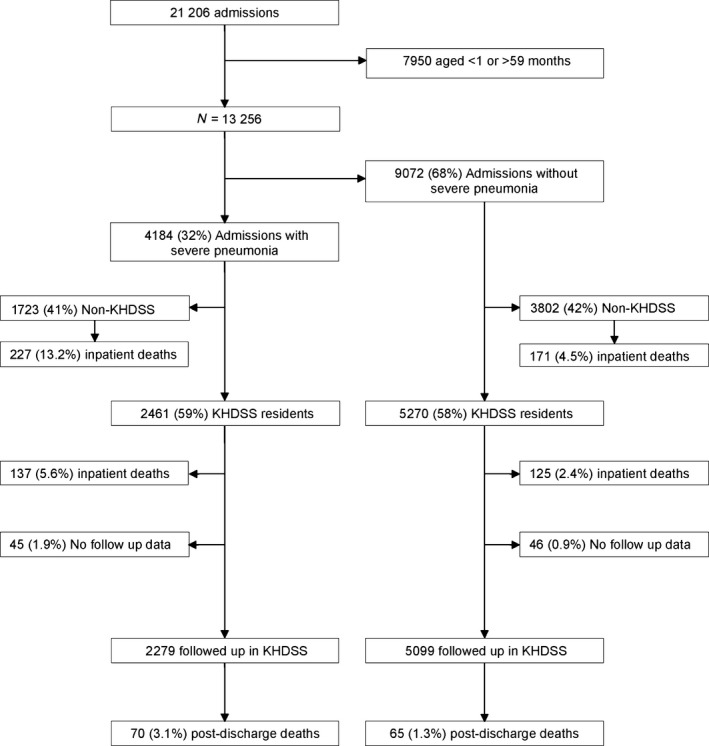
Recruitment and follow‐up.

Overall, 4184 children (1–59 months) were admitted with severe pneumonia, comprising 32% of all admissions within the study age range (Figure 1). Their median age (IQR) was 8.9 (4 to 19) months; 578 (14%) were hypoxic at admission, 1041 (25%) were severely malnourished, and 267 (6.4%) had a positive HIV antibody test (Table 1). Three hundred and sixty‐four (8.7%) children with severe pneumonia died in hospital (55% of inpatient deaths within this age range) compared with 296/8908 (3.3%) amongst all children admitted without severe pneumonia (age‐adjusted relative risk (RR) 2.6, 95% CI 2.3, 3.1). Within the KHDSS, children admitted with severe pneumonia had more severe disease and co‐morbidities than children admitted without severe pneumonia. There were 137/2461 (5.6%) inpatient deaths among children admitted with severe pneumonia compared to 125/5270 (2.4%) among children admitted without severe pneumonia within KHDSS [age‐adjusted relative risk [RR] 2.3, (95% CI 1.8, 2.9)].

**Table 1:** Patient characteristics at admission

	All severe pneumonia admissions (*n* = 4184)	Severe pneumonia admissions among KHDSS Residents (*n* = 2461)	Admission without severe pneumonia among KHDSS Residents (*n* = 5270)
Demographic characteristics^a^			
Age in months: median (IQR)	8.9 (4, 19)	9.3 (3.9, 20.4)	19.5 (11, 28.5)
Female	1846 (44)	1064 (43)	2297 (44)
Reported preterm/LBW	166 (4.0)	74 (3.0)	108 (2.1)
Hospitalization time (days): median (IQR)	4 (2, 6)	3 (2, 5)	3 (2, 6)
Previous hospital admission	109 (2.6)	69 (2.8)	128 (2.4)
Clinical characteristics at admission			
Hypoxia (SaO_2_ <90%)	578 (14)	304 (12)	73 (1.4)
Capillary refill >2 s	202 (4.8)	89 (3.6)	116 (2.2)
Impaired consciousness^b^	329 (7.9)	161 (6.5)	234 (4.4)
Wheezing	671 (16)	432 (18)	27 (0.5)
Cough for >14 days	185 (4.8)	68 (3.0)	117 (6.9)
Jaundice	34 (0.8)	15 (0.6)	73 (1.4)
Severe anaemia	114 (3.4)	59 (3.0)	174 (3.3)
Axillary temperature <36°C	112 (2.7)	71 (2.9)	172 (3.3)
Axillary temperature 36–39°C	3355 (80)	1988 (81)	4159 (79)
Axillary temperature >39°C	717 (17)	402 (16)	939 (18)
HIV antibody test positive	267 (6.4)	110 (4.5)	145 (2.8)
HIV test not performed	307 (7.3)	180 (7.3)	634 (12)
RSV test positive	805 (19)	485 (20)	9 (0.2)
RSV test not performed	1109 (27)	723 (29)	5146 (98)
Malaria slide positive	401 (9.6)	240 (9.8)	1203 (23)
Bacteraemia	189 (4.5)	113 (4.6)	308 (5.8)
Nutritional characteristics at admission			
WHZ Mean (sd)^c^	−1.1 (1.8)	−0.9 (1.7)	−1.1 (1.5)
HAZ Mean (sd)^d^	−1.6 (1.7)	−1.5 (1.6)	−1.7 (1.6)
WAZ Mean (sd)^e^	−1.9 (1.7)	−1.7 (1.6)	−1.7 (1.4)
HCAZ Mean (sd)^f^	−0.5 (1.7)	−0.4 (1.6)	−0.5 (1.5)
MUAC cm Median (IQR)^g^	13.0 (11.8, 14.0)	13.0 (12.0, 14.2)	14.0 (12.6, 14.9)
Kwashiorkor (nutritional oedema)	68 (1.6)	25 (1.0)	234 (4.5)
Outcome			
Inpatient death	364 (8.7)	137 (5.6)	126 (2.4)

^a^data given as *N* (%) unless otherwise indicated, IQR: interquartile range, LBW: low birthweight (<2500 g), ^b^conscious level classified as ‘prostrate’ or ‘unconscious’, RSV: Respiratory Syncytial Virus, ^e^115 missing, ^d^378 missing, ^c^454 missing, ^f^168 missing, sd: Standard deviation, ^g^161 missing, WHZ: weight for length/height z‐score, HAZ: length/height for age z‐score, WAZ: weight for‐age z‐score, HCAZ: head circumference for age z‐score, MUAC: Mid‐upper arm circumference, and KHDSS: Kilifi Health and Demographic Surveillance System.

Amongst children admitted with severe pneumonia, non‐KHDSS residents had more severe disease and more co‐morbidities than KHDSS residents (Table S1). Overall, 137/2461 (5.6%) KHDSS‐residents died in hospital compared to 227/1723 (13.2%) non‐KHDSS residents (RR 0.4, 95% CI 0.34, 0.52).

Two thousand two hundred and seventy‐nine KHDSS‐resident children with severe pneumonia were discharged alive and followed up for 1 year, giving 2163 child‐years, during which 70 (3.1%) children died (Figure 1). Twenty‐six (37%) of the 70 deaths occurred during a subsequent hospital admission. Post‐discharge deaths comprised 70/207 (34%) of all inpatient and post‐discharge deaths amongst KHDSS‐resident children admitted with severe pneumonia. Six (8.6%), 19 (27%), 31 (44%), and 52 (74%) post‐discharge deaths occurred within 7, 30, 90, and 180 days, respectively. The post‐discharge mortality rates from discharge to months 3, 6, and 12 were 144 (95% CI 101, 205), 65 (95% CI 49, 86), and 32 (95% CI 26, 41) deaths per 1000 child‐years, respectively.

**Figure 2:**
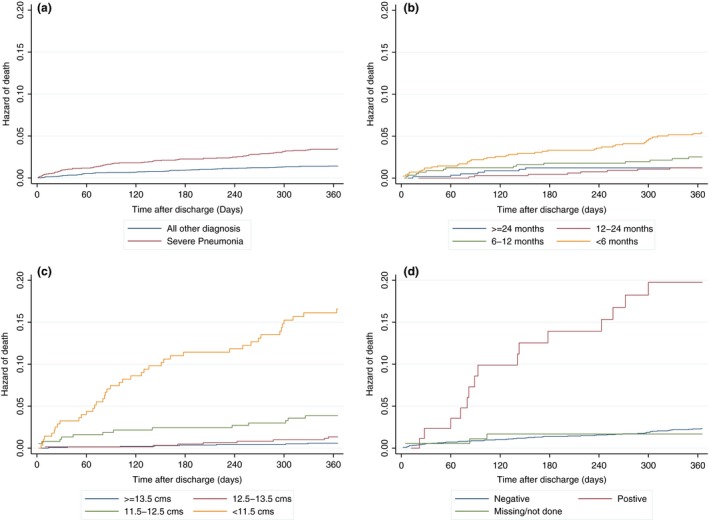
Hazards of post‐discharge death associated with (a) severe pneumonia vs. other diagnoses; (b) age at admission; (c) MUAC at admission; and (d) HIV status.

For comparison, of 5099 KHDSS‐resident children admitted during the same period without severe pneumonia, 65 (1.3%) children died within 1‐year post‐discharge (4898 child years): 13 (95% CI 10, 17) deaths per 1000 child years. Thus, severe pneumonia comprised 70/135 (52%) of all post‐discharge deaths. Children admitted with severe pneumonia had a higher risk of post‐discharge mortality than children admitted without severe pneumonia (age adjusted hazard ratio 2.50 (95% CI 1.17, 5.32) (Figure 2a).

Within the severe pneumonia cohort, young age, nutritional status, HIV status, and prolonged duration of hospitalisation, were associated with post‐discharge mortality (Table 2, Tables S2, S3, S4 and Figure 2b, c and d). Severe underweight (weight‐for‐age z‐score <−3) and a positive HIV test were present in 38 (54%) and 16 (23%) of the 70 children who died post‐discharge. Eight (11%) children who died had both severe underweight and a positive HIV test. Distance from the hospital was associated with post‐discharge mortality in the univariable model, but the effect was not evident in the multivariable model (Table 2, Tables S2, S3). We found interaction between HIV status and weight‐for‐height z‐score (*P* < 0.01) but no interaction with height‐for‐age z‐score (*P* = 0.42), weight‐for‐age z‐score (*P* = 0.31), or MUAC (*P* = 0.63). The effect of nutritional status on post‐discharge mortality was attenuated slightly after adjusting for HIV status (Table S4). There was no evidence of interaction between age and MUAC (*P* = 0.27), weight‐for‐height z‐score (*P* = 0.09) or HIV status (*P* = 0.47) on post‐discharge mortality. However, we found interaction between age and height‐for‐age z‐score (*P* < 0.01) on post‐discharge mortality. In the model with wasting and stunting, the predominant effect was from stunting (Table S3). The bootstrapped AUROCs for the final models were similar to the original models based on MUAC (Table 2), wasting and stunting, and weight‐for‐age z‐score (Table S3).

**Table 2:** Multivariable analysis of factors associated with 1‐year post‐discharge mortality among KHDSS residents (*n* = 2279)

(Child years of observation (cyo)=2163)	Number (Mortality rate per 1000cyo)	Adjusted HR (95% CI)
Demographic characteristics		
Age in months		
≥24 months	401 (12.4)	1.0 (Reference)
12–23 months	546 (13.8)	1.0 (0.1, 9.6)
6–11 months	519 (27.2)	5.8 (0.8, 40.5)
<6 months	813 (57.2)	4.8 (0.7, 34.1)
Female	976 (29.1)	0.5 (0.3, 1.1)
Reported preterm/LBW	64 (99.5)	0.7 (0.2, 2.8)
Residence distance from KCH per km	2117	1.0 (0.9, 1.1)
Duration of hospitalization per day	2276	1.1 (1.0, 1.2)
Clinical characteristics at admission		
Hypoxia (SaO_2_ <90%)	247 (87.9)	1.9 (0.7, 5.4)
Capillary refill >2 s	48 (188.3)	2.4 (0.5, 12.1)
Impaired consciousness^a^	103 (71.6)	1.1 (0.2, 7.8)
Wheezing	424 (9.6)	0.5 (0.1, 2.4)
Cough for >14 days	55 (92.7)	0.2 (0.1, 5.5)
Jaundice	13 (75.5)	12.5 (1.1, 13.7)
Severe anaemia (Hb <5 g/dL)	48 (42.7)	0.8 (0.1, 7.5)
Axillary temperature <36°C	58 (35.4)	0.3 (0.1, 2.8)
Axillary temperature 36 to 39°C	1858 (30.9)	1.0 (Reference)
Axillary temperature >39°C	363 (24.5)	1.1 (0.4, 3.0)
HIV antibody test negative	2035 (25.1)	1.0 (Reference)
HIV antibody test positive	85 (202.6)	6.5 (2.3, 18.4)
HIV test not performed	159 (17.3)	0.4 (0.1, 3.6)
RSV test negative	1174 (32.5)	1.0 (Reference)
RSV test positive	477 (13.1)	0.3 (0.1, 1.2)
RSV test not performed	628 (34.5)	2.7 (1.2, 6.3)
Malaria slide negative	2073 (32.6)	1.0 (Reference)
Malaria slide positive	206 (5.3)	0.5 (0.1, 5.2)
No bacteraemia	2187 (29.2)	1.0 (Reference)
Bacteraemia	92 (43.5)	0.8 (0.1, 5.2)
MUAC per cm	2218	0.6 (0.5, 0.8)
Year of admission		
2007	480 (27.0)	1.0 (Reference)
2008	381 (31.4)	0.9 (0.3, 3.1)
2009	392 (19.9)	0.5 (0.1, 2.1)
2010	392 (28.5)	0.7 (0.2, 2.5)
2011	319 (35.2)	1.7 (0.5, 5.3)
2012	315 (41.6)	1.8 (0.2, 15.7)
Model performance		
AIC		526.9
Raw AUC (95% CI)		0.91 (0.88, 0.95)
Bootstrapped AUC (95% CI)		0.92 (0.88, 0.96)

a conscious level classified as ‘prostrate’ or ‘unconscious’, RSV: Respiratory Syncytial Virus, MUAC: mid‐upper arm circumference, LBW: Low birthweight (<2.5 kg), AIC: Akaike information criterion, AUC: area under the receiver operating characteristics curve.

For individual anthropometric indices, the performance of MUAC and weight‐for‐age did not differ (Table S4), whilst weight‐for‐length/height and length/height‐for‐age performed less well than MUAC in predicting post‐discharge mortality.

Among the 1979 children with no SAM, a total of 14 (0.7%), 20 (1.0%), and 24 (1.2%) children died during months 3, 6, and 12 of follow‐up, respectively. While 22 (7.3%), 32 (11%), and 46 (15%) children died within months 3, 6, and 12 respectively among SAM children. MUAC <11.5 cm alone (indicative of severe malnutrition) was strongly associated with post‐discharge mortality (Figure 2c). Among children with MUAC <11.5 cm, 14.0% died within 1 year. This contrasts with non‐malnourished children with MUAC ≥13.5 cm, among whom 1.0% died within 1 year (adjusted HR 11.8, 95% CI 5.7, 24.5) (Table S4).

After adjusting for age, HIV status, and gender, the fractions of post‐discharge mortality attributable to MUAC <13.5 cm; weight‐for‐length/height z‐score <−1; weight‐for‐age z‐score <−1; and height/length‐for‐age z‐score <−1 were 52% (95% CI 37, 63); 13% (95% CI 3.2, 22); 57% (95% CI 42, 68); and 34% (95% CI 18, 46), respectively. The fraction of post‐discharge deaths accounted for by a positive HIV test was 11% (95% CI 3.3, 18) after adjusting for age, gender, and MUAC.

## Comment

Amongst Kenyan children (1–59 months), admitted to hospital, approximately a third of deaths in children admitted with severe pneumonia and followed up for 12 months occurred after discharge. The risk of post‐discharge mortality amongst children admitted with severe pneumonia was more than twice that of those admitted with other conditions. We had previously reported data from a large clinical trial showing a similar relationship between clinical syndrome at the index admission and post‐discharge mortality amongst HIV‐uninfected children with SAM,^29^ the current results extend this phenomenon to all admissions. Thus, admission with severe pneumonia can be regarded as an important marker of vulnerability, partly because it captures effects of common co‐morbidities. Malnutrition, rather than HIV, was the predominant risk factor for post‐discharge mortality, despite the provision of outpatient treatment services for malnutrition.

We found an inpatient case fatality ratio for severe pneumonia of 8.7%, which is much higher than the most recent regional estimate of case fatality for Africa, derived from 11 studies, of 3.9%.^30^ This was despite an increase in staffing, an ensured supply of essential drugs, and careful adherence to WHO treatment guidelines because of the presence of the research centre. These findings concord with the inpatient case fatality ratio of 9.8% reported at district hospitals throughout Malawi following an intervention to improve case management,^31^ and suggest the need to investigate new treatment strategies.

Nutritional status was the major driver of post‐discharge mortality. MUAC was an efficient single marker of mortality risk, performing better than weight‐for‐height z‐score, the traditional marker of acute malnutrition, including after adjustment for age and gender which are known confounders of MUAC.^25^, ^27^ It could be argued that because MUAC changes with age, it predominantly captures the youngest children, rather than nutritional status. However, mortality was far higher amongst all children with MUAC <11.5 cm than amongst those who were simply aged under 6 months (Figure 2b and c) and the relationship between MUAC and mortality appeared to be less confounded by age than other nutritional indices. Weight‐for‐age performed as well as MUAC in predicting post‐discharge mortality in our study and previously was identified as a strong predictor of mortality in the community.^32^ Weight‐for‐age is not currently used to define acute malnutrition, but could be used as an alternative indicator. Height‐for‐age z‐score (stunting) also predicted post‐discharge mortality (Table S4), possibly reflecting chronic disadvantage and ill‐health.

Our findings concur with data from Bangladesh^13^ in suggesting that children who died after admission were younger and more severely malnourished. The proportions (7.3% and 11%) of severely malnourished children with severe pneumonia who died within 3 and 6 months after discharge were also very similar to published data from Gambia and Bangladesh.^11^, ^13^ Likewise, amongst children without malnutrition, our finding of ~1% who died post‐discharge also was very similar to studies in Gambia and Bangladesh,^9^, ^11^ suggesting generalisability when stratified by nutritional status, despite the potential limitations of our study outlined below.

We observed that post‐discharge mortality was not associated with hypoxia or other signs of acute severity, as previously has been reported from The Gambia.^8^ This is likely to be because children with severe signs more often died during the inpatient phase. However, in Uganda, in a study where severe pneumonia was not strictly defined according to WHO criteria, two features of severity, oxygen saturation and coma score were associated with post‐discharge mortality after adjustment for HIV and nutritional status.^12^

The strengths of this study were its large sample size; systematically collected data at admission to hospital, including prospective anthropometry and HIV status; and a long duration of follow‐up by systematic census with limited missing outcomes. An important limitation is that it includes only a subset of KHDSS‐resident children at a single site. Non‐KHDSS resident children had a higher prevalence of predictive co‐morbidities, a lower level of access and a higher inpatient case fatality ratio that suggest that their post‐discharge mortality is likely to be higher than that of KHDSS resident children. Other sites in lower middle income countries (LMICs) may also differ in relation to factors including immunization, access to health care services, and the prevalence of HIV, and facilities for its treatment. We were not able to assess the impact of tuberculosis (TB) contact or antiretroviral therapy or co‐trimoxazole prophylaxis in post‐discharge mortality amongst HIV‐infected children. Our investigation of variables potentially associated with post‐discharge mortality was guided by prior reports, but could have resulted in over‐fitting.

An inherent limitation is that the clinical syndrome of severe pneumonia is sensitive rather than specific for radiologically defined pneumonia and death. It captures other respiratory tract infections such as bronchiolitis or tuberculosis,^33^ as well as respiratory distress due to sepsis or malaria. However, it reflects the diagnostic criteria typically used in similar settings. It is also possible that some children with severe pneumonia were excluded from our study if they did not exhibit the usual clinical signs of pneumonia, which may occur more frequently amongst malnourished children.^34^

Further research is needed to examine the mechanisms of post‐discharge mortality, including the roles of ability to access health care, maternal mental and physical health, HIV care, undetected TB and the extent to which nosocomial acquisition of resistant pathogens may contribute to the mortality observed.

## Conclusions

In rural Kenya, the majority of inpatient and post‐discharge child deaths were associated with severe pneumonia, often in children with underlying co‐morbidities. In this study and in prior studies, where assessed, anthropometric markers of malnutrition were consistently the principal driver of mortality. Post‐discharge mortality is under‐recognised within treatment guidelines and practice. Risk stratification and a better understanding of the mechanisms underlying post‐discharge mortality, especially for undernourished children, are needed to reduce mortality after treatment for pneumonia.

## Supporting information


**Table S1.** Patient profile at admission by KHDSS residence.
**Table S2.** Univariable analysis of factors associated with post‐discharge mortality.
**Table S3.** Multivariable analysis of factors associated with 1‐year post‐discharge mortality.
**Table S4.** Association between nutritional status and post‐discharge mortality.
