# Supplementary material for: Mortality after Inpatient Treatment for Severe Pneumonia in Children: a Cohort Study
Source: Paediatr Perinat Epidemiol. 2017 Mar 20;31(3):233–42. doi: 10.1111/ppe.12348 (PMC5434848; doi:10.1111/ppe.12348)
Supplement: Supplementary file 1 — Table S1. Patient profile at admission by KHDSS residence. Table S2. Univariable analysis of factors associated with post‐discharge mortality. Table S3. Multivariable analysis of factors associated with 1‐year post‐discharge mortality. Table S4. Association between nutritional status and post‐discharge mortality. [file PPE-31-233-s001.docx]

**Supplementary Material**

Table of Contents

[Supplementary Table 1: Patient profile at admission by KHDSS residence. 2](#_Toc469146689)

[Supplementary Table 2: Univariable analysis of factors associated with post-discharge mortality. 3](#_Toc469146690)

[Supplementary Table 3: Multivariable analysis of factors associated with one year post-discharge mortality. 4](#_Toc469146691)

[Supplementary Table 4: Association between nutritional status and post discharge mortality. 5](#_Toc469146692)

|  | | | **All admissions with severe pneumonia**  **(N=4184)** | | | **KHDSS Residents with severe pneumonia (N=2461)** | | | **Non-KHDSS Residents with severe pneumonia (N=1723)** | | | **Univariate P-value** | | |
| --- | --- | --- | --- | --- | --- | --- | --- | --- | --- | --- | --- | --- | --- | --- |
| **Demographic characteristics ^a^** | | | | | | | | | | | | | | |
| Age in months: median (IQR) | | | 8.9 (4-19) | | | 9.3 (3.9-20.4) | | | 8.3 (4-17) | | | <0.001 | | |
| Female | | | 1846 (44) | | | 1064 (43) | | | 782 (45) | | | 0.17 | | |
| Reported premature/LBW | | | 166 (4.0) | | | 74 (3.0) | | | 92 (5.3) | | | <0.001 | | |
| Hospitalization time (days): median (IQR) | | | 4 (2-6) | | | 3 (2-5) | | | 4 (2-7) | | | <0.001 | | |
| Previous hospital admission | | | 109 (2.6) | | | 69 (2.8) | | | 40 (2.3) | | | 0.34 | | |
| **Clinical characteristics at admission** | | | | | | | | | | | | | | |
| Hypoxia (SaO_2_ <90%) | | | 578 (14) | | | 304 (12) | | | 274 (16) | | | 0.001 | | |
| Capillary refill >2 seconds | | | 202 (4.8) | | | 89 (3.6) | | | 113 (6.6) | | | <0.001 | | |
| Impaired consciousness ^b^ | | | 329 (7.9) | | | 161 (6.5) | | | 168 (9.8) | | | <0.001 | | |
| Wheezing | | | 671 (16) | | | 432 (18) | | | 239 (14) | | | 0.001 | | |
| Cough for >14 days | | | 185 (4.8) | | | 68 (3.0) | | | 117 (7.6) | | | <0.001 | | |
| Jaundice | | | 34 (0.8) | | | 15 (0.6) | | | 19 (1.1) | | | 0.08 | | |
| Severe anaemia | | | 114 (3.4) | | | 59 (3.0) | | | 55 (4.0) | | | 0.22 | | |
| Axillary temperature <36°C | | | 112 (2.7) | | | 71 (2.9) | | | 41 (2.4) | | | 0.18 | | |
| Axillary temperature 36-39°C | | | 3355 (80) | | | 1988 (81) | | | 1367 (79) | | |  |  |  |
| Axillary temperature >39°C | | | 717 (17) | | | 402 (16) | | | 315 (18) | | |  |  |  |
| HIV antibody test positive | | | 267 (6.4) | | | 110 (4.5) | | | 157 (9.1) | | | <0.001 | | |
| HIV test not preformed | | | 307 (7.3) | | | 180 (7.3) | | | 127 (7.4) | | |  |  |  |
| RSV test positive | | | 805 (19) | | | 485 (20) | | | 320 (19) | | | 0.03 | | |
| RSV test not preformed | | | 1109 (27) | | | 723 (29) | | | 386 (22) | | |  |  |  |
| Malaria slide positive | | | 401 (9.6) | | | 240 (9.8) | | | 161 (9.3) | | | 0.66 | | |
| Bacteraemia | | | 189 (4.5) | | | 113 (4.6) | | | 76 (4.4) | | | 0.78 | | |
| **Nutritional characteristics at admission** | | | | | | | | | | | | | | |
| WHZ Mean (sd) ^c^ | | | -1.1 (1.8) | | | -0.9 (1.7) | | | -1.3 (1.8) | | | <0.001 | | |
| HAZ Mean (sd) ^d^ | | | -1.6 (1.7) | | | -1.5 (1.6) | | | -1.8 (1.8) | | | <0.001 | | |
| WAZ Mean (sd) ^e^ | | | -1.9 (1.7) | | | -1.7 (1.6) | | | -2.2 (1.8) | | | <0.001 | | |
| HCAZ Mean (sd) ^f^ | | | -0.5 (1.7) | | | -0.4 (1.6) | | | -0.6 (1.8) | | | <0.001 | | |
| MUAC cm Median (IQR) ^g^ | | | 13.0 (11.8-14.0) | | | 13.0 (12.0-14.2) | | | 12.5 (11.0-14.0) | | | <0.001 | | |
| Kwashiorkor (nutritional oedema) | | | 68 (1.6) | | | 25 (1.0) | | | 43 (2.5) | | | <0.001 | | |
| **Outcome** | | | | | | | | | | | | | | |
| Inpatient death | | | 364 (8.7) | | | 137 (5.6) | | | 227 (13.2) | | | <0.001 | | |
| Supplementary Table 1: **Patient profile at admission by KHDSS residence**. | | | | | | | | | | | | | | |
| ^a^ data given as N (%) unless otherwise indicated, IQR: interquartile range, LBW: low birth weight (<2500grams), ^b^ conscious level classified as ‘prostrate’ or ‘unconscious’, RSV: Respiratory Syncytial Virus, ^e^ 115 missing, ^d^ 378 missing, ^c^ 454 missing, ^f^ 168 missing, sd: Standard deviation, ^g^ 161 missing, WHZ: weight for length/height z-score, HAZ: length/height for age z-score, WAZ: weight for-age z-score, HCAZ: head circumference for age z-score, MUAC: Mid-upper arm circumference, and KHDSS: Kilifi Health and Demographic Surveillance System | | | | | | | | | | | | | | |

| **(CYO=2,163)** | | **N=2279** | | **Crude HR** | | **95% CI** | | **P-value** | |
| --- | --- | --- | --- | --- | --- | --- | --- | --- | --- |
| **Demographic characteristics** | | | | | | | | | |
| >=24 months | | 401 | | 1·0 | | Reference | |  | |
| 12-24 months | | 546 | | 2.38 | | 0.78 to 7.29 | | 0.13 | |
| 6-12 months | | 519 | | 3.10 | | 1.04 to 9.27 | | 0.04 | |
| <6 months | | 813 | | 4.64 | | 1.65 to 13.0 | | 0.004 | |
| Distance from KCH (KMs) | | 2117 | | 1.04 | | 1.02 to 1.07 | | 0.002 | |
| Reported premature/LBW | | 64 | | 2.75 | | 1.11 to 6.83 | | 0.03 | |
| Hospitalization time (days) | | 2276 | | 1.07 | | 1.06 to 1.09 | | <0.001 | |
| Previous admission | | 62 | | 2.24 | | 0.82 to 6.14 | | 0.12 | |
| **Clinical characteristics at admission** | | | | | | | | | |
| Hypoxia (SaO_2_ <90%) | | 247 | | 2.73 | | 1.58 to 4.71 | | <0.001 | |
| Capillary refill >2 seconds | | 48 | | 3.87 | | 1.56 to 9.61 | | 0.004 | |
| Impaired consciousness^a^ | | 103 | | 1.29 | | 0.47 to 3.54 | | 0.62 | |
| Wheezing | | 424 | | 0.33 | | 0.13 to 0.81 | | 0.02 | |
| Cough for >14 days | | 55 | | 2.67 | | 0.97 to 7.34 | | 0.06 | |
| Jaundice | | 13 | | 2.53 | | 0.35 to 18.2 | | 0.36 | |
| Severe anaemia | | 48 | | 1.39 | | 0.34 to 5.70 | | 0.65 | |
| Axillary temperature <36°C | | 58 | | 1.06 | | 0.26 to 4.33 | | 0.94 | |
| Axillary temperature 36-39°C | | 1858 | | 1.0 | | Reference | |  | |
| Axillary temperature >39°C | | 363 | | 0.67 | | 0.32 to 1.41 | | 0.29 | |
| HIV antibody test negative | | 2035 | | 1.0 | | Reference | |  | |
| HIV antibody test positive | | 85 | | 6.05 | | 3.30 to 11.1 | | <0.001 | |
| HIV test not performed | | 159 | | 0.71 | | 0.22 to 2.28 | | 0.57 | |
| RSV test negative | | 1174 | | 1.0 | | Reference | |  | |
| RSV test positive | | 477 | | 0.22 | | 0.07 to 0.71 | | 0.01 | |
| RSV test not performed | | 628 | | 1.88 | | 1.16 to 3.03 | | 0.01 | |
| Malaria slide negative | | 2073 | | 1.0 | | Reference | |  | |
| Malaria slide positive | | 206 | | 0.45 | | 0.14 to 1.42 | | 0.17 | |
| No bacteraemia | | 2187 | | 1.0 | | Reference | |  | |
| Bacteraemia | | 92 | | 1.87 | | 0.75 to 4.64 | | 0.18 | |
| **Nutritional characteristics at admission** | | | | | | | | | |
| Weight for-age per Z-score | | 2248 | | 0.56 | | 0.50 to 0.63 | | <0.001 | |
| Weight-for-Height per Z-score | | 2115 | | 0.65 | | 0.56 to 0.75 | | <0.001 | |
| Length-for-age per Z-score | | 2137 | | 0.67 | | 0.59 to 0.75 | | <0.001 | |
| MUAC in cm | | 2218 | | 0.57 | | 0.52 to 0.64 | | <0.001 | |
| Kwashiorkor (nutritional oedema) | | 15 | | 2.32 | | 0.32 to 16.7 | | 0.40 | |
| Supplementary Table 2: **Univariable analysis of factors associated with post-discharge mortality**. | | | | | | | | | |
| LBW: low birth weight (<2500grams)**,** ^a^ conscious level classified as ‘prostrate’ or ‘unconscious’, RSV: Respiratory Syncytial Virus, MUAC: Mid-upper arm circumference. | | | | | | | | | |

| **(CYO=2,163)** | | **N** | | **Adjusted HR 95% CI P-value** | | | | **Adjusted HR 95% CI P-value** | | | |
| --- | --- | --- | --- | --- | --- | --- | --- | --- | --- | --- | --- |
| **Demographic characteristics** | | | | | | | | | | | |
| >=24months | | 401 | | 1.0 | Reference |  | | 1.0 | Reference |  | |
| 12-24months | | 546 | | 1.17 | 0.17 to 8.10 | 0.87 | | 0.98 | 0.14 to 7.08 | 0.99 | |
| 6-12months | | 519 | | 12.4 | 2.33 to 66.0 | 0.003 | | 6.10 | 1.24 to 30.1 | 0.03 | |
| <6months | | 813 | | 16.2 | 2.87 to 91.5 | 0.002 | | 10.01 | 2.24 to 44.7 | 0.003 | |
| Female | | 976 | | 0.65 | 0.28 to 1.47 | 0.30 | | 0.59 | 0.27 to 1.27 | 0.18 | |
| Distance from KCH (KMs) | | 2117 | | 1.02 | 0.99 to 1.06 | 0.18 | | 1.04 | 0.99 to 1.08 | 0.06 | |
| Reported premature/LBW | | 64 | | 0.46 | 0.05 to 4.20 | 0.50 | | 0.69 | 0.15 to 3.09 | 0.63 | |
| Hospitalization time (days) | | 2276 | | 1.09 | 1.03 to 1.15 | 0.003 | | 1.07 | 1.01 to 1.14 | 0.02 | |
| Previous admission | | 62 | | 0.62 | 0.17 to 2.22 | 0.46 | | 0.77 | 0.25 to 2.38 | 0.65 | |
| **Clinical characteristics at admission** | | | | | | | | | | | |
| Hypoxia (SaO_2_ <90%) | | 247 | | 1.32 | 0.37 to 4.70 | 0.67 | | 1.37 | 0.46 to 4.12 | 0.57 | |
| Capillary refill >2 seconds | | 48 | | 1.42 | 0.19 to 10.4 | 0.73 | | 1.63 | 0.32 to 8.30 | 0.55 | |
| Impaired consciousness^a^ | | 103 | | 1.62 | 0.14 to 18.3 | 0.70 | | 0.88 | 0.11 to 6.83 | 0.90 | |
| Wheezing | | 424 | | 0.45 | 0.10 to 2.06 | 0.30 | | 0.43 | 0.09 to 2.05 | 0.29 | |
| Cough for >14 days | | 55 | | 0.05 | 0.01 to 3.75 | 0.17 | | 0.25 | 0.01 to 7.91 | 0.43 | |
| Jaundice | | 13 | | 14.5 | 2.20 to 94.9 | 0.005 | | 11.8 | 1.45 to 96.0 | 0.02 | |
| Severe anaemia (Hb <5g/dl) | | 48 | | 0.52 | 0.05 to 4.95 | 0.57 | | 0.73 | 0.07 to 7.25 | 0.79 | |
| Axillary temperature <36°C | | 58 | | 0.38 | 0.05 to 3.08 | 0.36 | | 0.50 | 0.07 to 3.79 | 0.50 | |
| Axillary temperature 36 to 39°C | | 1858 | | 1.0 | Reference |  | | 1.0 | Reference |  | |
| Axillary temperature >39°C | | 363 | | 0.81 | 0.27 to 2.34 | 0.70 | | 1.34 | 0.53 to 3.35 | 0.54 | |
| HIV antibody test negative | | 2035 | | 1.0 | Reference |  | | 1.0 | Reference |  | |
| HIV antibody test positive | | 85 | | 6.13 | 2.33 to 16.2 | <0.001 | | 5.14 | 1.63 to 16.2 | 0.005 | |
| HIV test not performed | | 159 | | 0.34 | 0.05 to 2.55 | 0.30 | | 0.49 | 0.05 to 4.65 | 0.53 | |
| RSV test negative | | 1174 | | 1.0 | Reference |  | | 1.0 | Reference |  | |
| RSV test positive | | 477 | | 0.38 | 0.10 to 1.46 | 0.16 | | 0.38 | 0.09 to 1.48 | 0.16 | |
| RSV test not performed | | 628 | | 2.58 | 1.10 to 6.05 | 0.03 | | 2.88 | 1.23 to 6.77 | 0.02 | |
| Malaria slide negative | | 2073 | | 1.0 | Reference |  | | 1.0 | Reference |  | |
| Malaria slide positive | | 206 | | 0.57 | 0.04 to 6.58 | 0.65 | | 0.53 | 0.05 to 5.17 | 0.58 | |
| No bacteraemia | | 2187 | | 1.0 | Reference |  | | 1.0 | Reference |  | |
| Bacteraemia | | 92 | | 0.47 | 0.12 to 1.77 | 0.26 | | 1.16 | 0.26 to 5.14 | 0.85 | |
| Weight-for-height per Z-score | | 2115 | | 0.81 | 0.63 to 1.03 | 0.09 | |  |  |  | |
| Length-for-age per Z-score | | 2137 | | 0.53 | 0.40 to 0.69 | <0.001 | |  |  |  | |
| Weight-for-age per Z-score | | 2248 | |  |  |  | | 0.61 | 0.50 to 0.76 | <0.001 | |
| Y**ear of admission** | | | | | | | | | | | |
| 2007 | | 480 | | 1.0 | Reference |  | | 1.0 | Reference |  | |
| 2008 | | 381 | | 0.87 | 0.29 to 2.61 | 0.80 | | 1.04 | 0.36 to 2.99 | 0.95 | |
| 2009 | | 392 | | 0.62 | 0.15 to 2.53 | 0.50 | | 0.59 | 0.16 to 2.15 | 0.43 | |
| 2010 | | 392 | | 0.64 | 0.17 to 2.42 | 0.52 | | 0.53 | 0.14 to 1.99 | 0.35 | |
| 2011 | | 319 | | 1.66 | 0.55 to 5.01 | 0.37 | | 1.30 | 0.37 to 4.50 | 0.68 | |
| 2012 | | 315 | | 1.17 | 0.11 to 12.0 | 0.89 | | 1.52 | 0.13 to 18.1 | 0.74 | |
| **Model performance** | | | | | | | | | | | |
| AIC 451.5 529.9 | | | | | | | | | | | |
| Raw AUC (95% CI) 0.91 (0.88 to 0.95) 0.92 (0.89 to 0.95) | | | | | | | | | | | |
| Bootstrapped AUC (95% CI) 0.92 (0.87 to 0.96) 0.92 (0.89 to 0.95) | | | | | | | | | | | |
| Supplementary Table 3: Multivariable analysis of factors associated with one year post-discharge mortality. | | | | | | | | | | | |
| CYO: Child years of observation, LBW: Low birth weight (<2500g), ^a^ conscious level classified as ‘prostrate’ or ‘unconscious’. RSV: Respiratory Syncytial Virus, AIC: Akaike information criterion, AUC: area under the receiver operating characteristic curve | | | | | | | | | | | |

| **(CYO=2,163)** | | **N** | | | **Died** | | **% died** | | **Crude HR** | | **95% CI** | | **Adjusted HR^a^** | | **95% CI** | |
| --- | --- | --- | --- | --- | --- | --- | --- | --- | --- | --- | --- | --- | --- | --- | --- | --- |
| **Weight for length/height Z-score** | | | | | | | | | | | | | | | | |
| >=-1 | | 1282 | | | 26 | | 2.0 | | 1.0 | | Reference | | 1.0 | | Reference | |
| -1 to -2 | | 531 | | | 18 | | 3.4 | | 1.68 | | 0.92 to 3.06 | | 2.03 | | 1.11 to 3.73 | |
| -2 to -3 | | 282 | | | 8 | | 2.8 | | 1.41 | | 0.64 to 3.11 | | 1.61 | | 0.72 to 3.58 | |
| <-3 | | 184 | | | 18 | | 9.8 | | 4.98 | | 2.73 to 9.09 | | 4.45 | | 2.40 to 8.26 | |
| **Model performance** | | | | | | | | | | | | | | | | |
| Raw AUC (95% CI) | |  | | | | | | | | | | | 0.74 (0.68 to 0.80) | | | |
| Bootstrapped AUC (95% CI) | |  |  |  |  |  |  |  |  |  |  |  | 0.76 (0.71 to 0.80) | | | |
| **Length/height for age Z-score** | | | | | | | | | | | | | | | | |
| >=-1 | | 992 | | | 15 | | 1.5 | | 1.0 | | Reference | | 1.0 | | Reference | |
| -1 to -2 | | 567 | | | 12 | | 2.1 | | 1.40 | | 0.66 to 3.00 | | 1.48 | | 0.69 to 3.17 | |
| -2 to -3 | | 447 | | | 19 | | 4.3 | | 2.85 | | 1.45 to 5.61 | | 3.26 | | 1.64 to 6.45 | |
| <-3 | | 273 | | | 24 | | 8.8 | | 6.07 | | 3.18 to 11.6 | | 5.94 | | 3.07 to 11.5 | |
| **Model performance** | |  | | | | | | | | | | |  | | | |
| Raw AUC (95% CI) | |  | | | | | | | | | | | 0.74 (0.69 to 0.81) | | | |
| Bootstrapped AUC (95% CI) | |  |  |  |  |  |  |  |  |  |  |  | 0.76 (0.72 to 0.79) | | | |
| **Weight-for-age Z-score** | |  | | |  | |  | |  | |  | |  | | | |
| ≥-1 | | 838 | | | 6 | | 0.7 | | 1.0 | | Reference | | 1.0 | | Reference | |
| -1 to -2 | | 643 | | | 13 | | 2.0 | | 2.86 | | 1.09 to 7.52 | | 3.18 | | 1.21 to 8.39 | |
| -2 to -3 | | 460 | | | 13 | | 2.8 | | 3.98 | | 1.51 to 10.5 | | 4.67 | | 1.77 to 12.3 | |
| <-3 | | 338 | | | 38 | | 11.2 | | 16.8 | | 7.09 to 39.7 | | 16.07 | | 6.70 to 38.5 | |
| **Model performance** | | | | | | | | | | | | | | | | |
| Raw AUC (95% CI) | |  | | | | | | | | | | | 0.81 (0.76 to 0.86) | | | |
| Bootstrapped AUC (95% CI) | |  |  |  |  |  |  |  |  |  |  |  | 0.82 (0.78 to 0.86) | | | |
| **MUAC in cm** | | | | | | | | | | | | | | | | |
| >13.5 | | | 1089 | | 11 | | 1.0 | | 1.0 | | Reference | | 1.0 | | Reference | |
| 12.5-13.5 | | | 541 | | 7 | | 1.3 | | 1.28 | | 0.50 to 3.30 | | 1.22 | | 0.47 to 3.17 | |
| 11.5-12.5 | | | 356 | | 11 | | 3.1 | | 3.10 | | 1.35 to 7.16 | | 2.71 | | 1.14 to 6.44 | |
| <11.5 | | | 293 | | 41 | | 14.0 | | 14.9 | | 7.67 to 29.0 | | 11.8 | | 5.67 to 24.5 | |
| **Model performance** | | | | | | | | | | | | | | | | |
| Raw AUC (95% CI) | | |  | | | | | | | | | | 0.81 (0.76 to 0.87) | | | |
| Bootstrapped AUC (95% CI) | | |  |  |  |  |  |  |  |  |  |  | 0.80 (0.77 to 0.84) | | | |
| Supplementary Table 4: Association between nutritional status and post discharge mortality. | | | | | | | | | | | | | | | | |
| ^a^ Adjusted for age, gender and HIV, MUAC: Mid-upper arm circumference, AUC: area under the receiver operating characteristic curve. | | | | | | | | | | | | | | | | |
